# Iron(III)–Tropolone Complex as a Topical Agent Against Drug-Resistant MRSA Skin Infections

**DOI:** 10.3390/antibiotics15030298

**Published:** 2026-03-14

**Authors:** Nalin Abeydeera, Guanyu Chen, Khalil Zarea, Bishnu D. Pant, Bogdan M. Benin, Kalpani M. Ratnayake, Min-Ho Kim, Woo Shik Shin, Songping D. Huang

**Affiliations:** 1 Department of Chemistry and Biochemistry, Kent State University, Kent, OH 44240, USA; nkekiriw@kent.edu (N.A.); gchen10@kent.edu (G.C.); bpant@kent.edu (B.D.P.); 2 Department of Physical Sciences, Chemistry Division, College of Science, Jazan University, P.O. Box. 114, Jazan 45142, Saudi Arabia; kmudarma@kent.edu; 3 Cupron Performance Additives, Inc., Richmond, VA 23231, USA; bbenin@cupron.com; 4 Department of Earth Sciences, Kent State University, Kent, OH 44240, USA; kalpanimanurangi@gmail.com; 5 Department of Biological Sciences, Kent State University, Kent, OH 44240, USA; mkim15@kent.edu; 6 Department of Pharmaceutical Sciences, College of Pharmacy and Health Sciences, Fairleigh Dickinson University, Florham Park, NJ 07932, USA

**Keywords:** Fe-based antimicrobials, methicillin-resistant *Staphylococcus aureus*, metal chelation, Fenton reaction, bacterial iron metabolism

## Abstract

**Background/Objectives**: The widespread use of mupirocin and fusidic acid for the treatment and decolonization of *Staphylococcus aureus* (SA) skin infections has led to a rapid emergence of resistant strains, limiting the effectiveness of the few topical agents currently available for clinical use. **Methods:** In this study, we evaluate Fe(tropo)_3_, a neutral and lipophilic iron(III)–tropolone complex, as a non-antibiotic topical antimicrobial candidate for the management of drug-resistant SA skin and soft tissue infections. **Results:** Fe(tropo)_3_ exhibits potent in vitro activity against methicillin-susceptible SA, methicillin-resistant SA (MRSA), vancomycin-intermediate SA, and strains with high-level resistance to mupirocin and fusidate, with minimum inhibitory concentrations of 2 µg/mL across all tested isolates. The compound effectively penetrates bacterial cells, induces intracellular iron accumulation, and triggers dose-dependent reactive oxygen species generation, resulting in rapid bacterial killing and significant antibiofilm activity. Importantly, Fe(tropo)_3_ shows a slower development of resistance compared with ciprofloxacin and displays synergistic activity with oxacillin against MRSA. When formulated as a 1% topical ointment, Fe(tropo)_3_ significantly reduces bacterial burden in a murine excisional wound infection model, achieving a 98% ± 1% reduction in SA load without detectable hemolysis or skin irritation. **Conclusions:** These pilot study results support Fe(tropo)_3_ as a clinically relevant, mechanism-distinct topical antimicrobial with potential utility in settings where resistance to existing topical antibiotics compromises standard care.

## 1. Introduction

*Staphylococcus aureus* (SA) is a versatile Gram-positive pathogen responsible for infections from minor skin lesions to fatal systemic diseases like endocarditis and toxic shock syndrome [1]. The rise in multidrug-resistant SA strains, coupled with their capacity to evade attack by the host innate immune system, has made treating methicillin-resistant SA (MRSA) infections, including even SSTIs, increasingly difficult [2,3,4,5,6]. It has been estimated that approximately 30% of people are colonized by SA on the skin and in the nares. With both hospital- and community-acquired SSTIs by MRSA on the rise, the need for nasal MRSA decolonization and treating MRSA SSTIs has never been greater. Hence, the lack of progress in the development of new effective topical antibiotics against MRSA is a great concern. Among over 100 different commercial antibiotics, only a few are suitable for use as topical antibiotics to treat SSTIs. These include bacitracin, neomycin, polymyxin B, mupirocin, and fusidate (i.e., fusidic acid), but only the latter two are effective against MRSA [7,8]. There are, however, an increasing number of MRSA strains that have developed resistance to mupirocin [9,10,11]. Similarly, fusidate has never been used as a topical monotherapy for treating MRSA SSTIs because the development of resistance to this antibiotic has a very low genetic barrier, requiring only a single-point mutation [12,13]. It should be noted that fusidate has never been approved for use as a topical antibiotic in the United States, although it has long been available in Europe [14,15].

In the discovery and development of new generations of topical antibiotics that are active against MRSA, it is highly desirable to explore novel antimicrobial modes of action to target the cellular or molecular components and pathways that have not been targeted by conventional antibiotics. For instance, there are approximately 200 conserved essential proteins in bacteria that can be potential antimicrobial targets, but current successful antibiotics hit about 40 of such targets in only three different pathways, i.e., the ribosome, cell wall synthesis, and DNA gyrase or DNA topoisomerase [16]. Accordingly, bacteria have evolved a variety of defense mechanisms to escape the attacks by the existing antibiotics to these targets and pathways, using the enzymatic drug modifications, enhanced efflux pump activity, or target site resistance mutations [17,18,19].

In this regard, metal compounds provide a large pool of molecules that can potentially access some of the new targets that current commercial antibiotics are unable to access [20,21,22]. Among all the bio-essential metals, iron is one of the most cytotoxic ones due to its redox activity that can trigger the intracellular Fenton reaction to produce reactive oxygen species (ROS), which may result in cell death. Consequently, cellular iron uptake in bacteria is tightly regulated via siderophores and/or heme-uptake systems, making the stray iron difficult to cross the cell membrane [23,24,25,26].

**Scheme 1:**
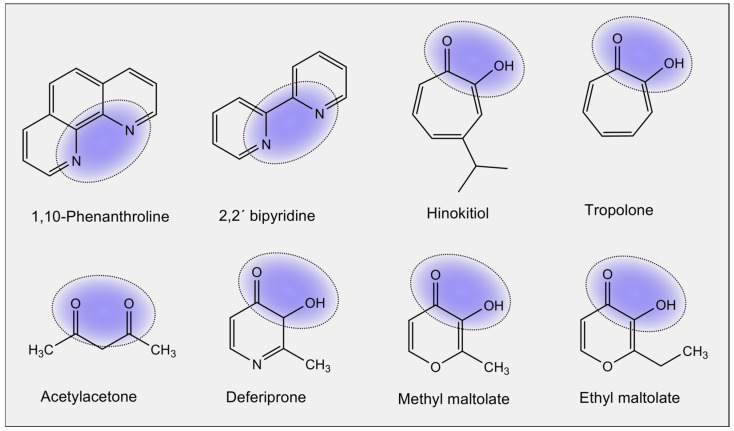
Bidentate chelating ligands containing hard Lewis donor atoms (O and/or N) were explored in this study, which provided key insights and led to the identification of Fe(tropo)_3_ as the most potent antimicrobial agent against SA.

In order to harness such cytotoxicity to kill bacteria, this obstacle needs to be overcome. Here, we investigated a series of charge-neutral, octahedral Fe(III) coordination complexes formed from bidentate ligands bearing the hard Lewis donor atoms oxygen (O) and/or nitrogen (N) donor atoms, with the aim of perturbing bacterial iron homeostasis and promoting iron transportation across the cell membrane as a strategy for the development of iron-based antimicrobial agents. This approach is based on the idea that the formation of *D*_3_–symmetric Fe(III) complexes reduces overall molecular polarity and masks the ionic nature of the metal center, thereby enhancing lipophilicity and facilitating transport across the bacterial cell membrane. Among various chelating ligands (see Scheme 1) we have examined for such purposes, Fe(hinok)_3_–the Fe(III) complex formed with the naturally occurring tropolone derivative hinokitiol (i.e., 2-hydroxy-4-isopropyl-2,4,6-cycloheptatrien-1-one; hinok)- exhibits the highest antimicrobial activity [27] (Table 1 and Figure S4).

To further extend this strategy toward the development of iron-based antimicrobial agents, we evaluated the antimicrobial activity of Fe(tropo)_3_, the Fe(III) complex derived from tropolone. Notably, despite the lower lipophilicity of tropolone (LogP = −0.34) relative to hinokitiol (LogP = 0.59), Fe(tropo)_3_ displayed comparable minimum inhibitory concentration (MIC) values against MSSA, suggesting that antimicrobial activity is not solely governed by ligand lipophilicity. In this publication, we show that due to the high lipophilicity, annulled molecular polarity, and idealized *D*_3_ symmetry, Fe(tropo)_3_ can readily penetrate the cell membrane to produce potent antimicrobial activity that is comparable to that of Fe(hinok)_3_ against the Gram-positive SA bacteria. In contrast, Fe(tropo)_3_ exhibits an improved resistance-development profile compared with Fe(hinok)_3_ [27] and is also capable of overcoming resistance in mupirocin- and fusidic acid–resistant *S. aureus* mutant strains. These results highlight the potential of Fe(tropo)_3_ as a topical antimicrobial agent for wound-site *S. aureus* decolonization and for the treatment of *S. aureus*-associated skin and soft tissue infections (SSTIs), where high-level resistance to mupirocin and fusidate has become increasingly prevalent. We also provide experimental evidence to show that the antimicrobial mode of action by Fe(tropo)_3_ is mainly attributable to the iron-mediated intracellular ROS generation.

## 2. Results and Discussion

### 2.1. Synthesis and Characterization of Fe(tropo)_3_

**Figure 1:**
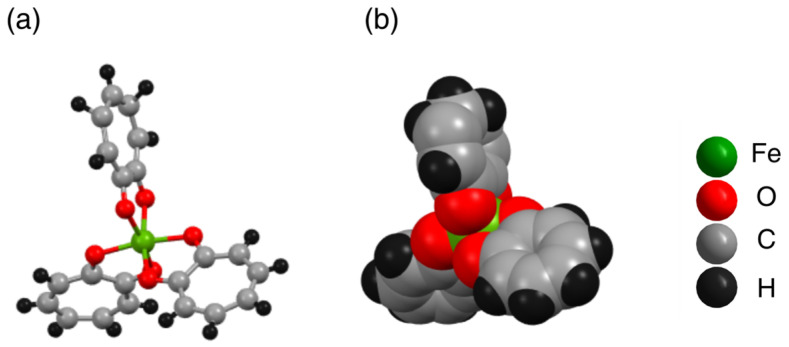
Representation of the molecular structure of Fe(tropo)_3_ depicted by the stick-and-ball model (**a**) and by the space-filling model (**b**).

Tropolone (3 mmol, 0.366 g) was dissolved in ethanol (20 mL), and a solution of anhydrous iron chloride (1 mmol, 0.162 g) in ethanol (5 mL) was added dropwise. The resulting mixture was stirred at room temperature for 3 h. The solid product was collected by filtration, washed with cold ethanol (3 × 5 mL), and dried under a vacuum oven (Scheme S1). Spectroscopic characterization, including elemental analysis (Table S1), UV-Vis (Figure S2), FT-IR (Figure S3), and X-ray powder diffraction (PXRD) (Figure S4), indisputably established the identity of the product as Fe(tropo)_3_ with purity ≥ 98%. The crystal structure reveals that Fe^3+^ is fully encapsulated by six oxygen (O) donor atoms from three tropolone ligands, masking the ionic character of the metal center (Figure 1a,b). This neutral Fe(III) complex adopts an octahedral geometry with idealized *D*_3_ symmetry, forming a racemic mixture of two enantiomers, which were not separated for the bioactivity studies.

### 2.2. In Vitro Antibacterial Activity Against Different Strains of SA Bacteria

Given the Fe(tropo)_3_’s potent antibacterial activity against methicillin-sensitive SA (MSSA; Table 1), Fe(tropo)_3_ was further evaluated against a panel of drug-resistant Gram-positive *S. aureus* strains using the broth microdilution method following Clinical and Laboratory Standards Institute (CLSI) guidelines [28]. The bacterial strains evaluated included a methicillin-susceptible *S. aureus* strain (MSSA), two methicillin-resistant strains (MRSA^α^ and MRSA^β^), and a vancomycin-intermediate *S. aureus* strain (VISA). In addition, laboratory-generated mutant MRSA^α^ strains resistant to mupirocin [MRSA^α(^^mupR)^], fusidate [MRSA^α(fusR)^], and both antibiotics [MRSA^α(mupR+fusR^] were obtained by stepwise exposure to increasing concentrations of antibiotics, as described in the methods section (Figure S5). The results show that Fe(tropo)_3_ exhibits potent antimicrobial activity against all seven bacterial strains, as revealed by the MIC values summarized in Table 2 and Figure S6.

**Figure 2:**
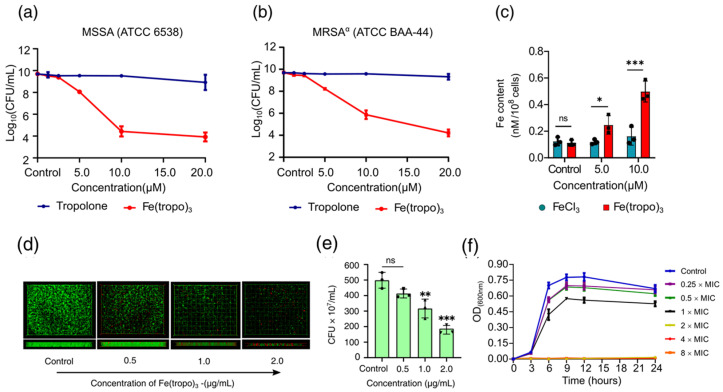
Antibacterial activity of Fe(tropo)_3_ against MSSA (**a**) and MRSA^α^ (**b**). Cellular uptake of Fe(tropo)_3_ in MRSA^α^ was quantified by measuring intracellular Fe levels in bacterial lysates (**c**). Representative top-view and side-view fluorescence images of MRSA^α^ biofilms treated with three different concentrations of Fe(tropo)_3_ (**d**). Quantification of bacterial growth inhibition in biofilm-derived MRSA (**e**). Time–kill kinetics of Fe(tropo)_3_ against MRSA^α^ at the indicated concentrations over 24 h (**f**). Data are shown as mean ± SD (*n* = 3 independent biological replicates). Statistical significance was determined as follows: * *p* < 0.05, ** *p* < 0.01, *** *p* < 0.001; ns, not significant.

To further confirm the enhancement effect of antibacterial activity through Fe(III) complexation with tropolone, we determined the antibacterial activity of Fe(tropo)_3_ against MSSA and MRSA^α^ using colony-forming unit (CFU) enumeration. Untreated cells served as controls, and tropolone alone was tested in parallel at concentrations three times higher than Fe(tropo)_3_, accounting for its three ligands. Fe(tropo)_3_ reduced MSSA CFUs by 1.5 log at 5.0 µM (2.0 µg/mL; 1× MIC), 5 log at 10.0 µM (4.0 µg/mL; 2× MIC), and 6 log at 20.0 µM (8.0 µg/mL; 4× MIC) (Figure 2a). In contrast, tropolone alone showed minimal activity, indicating that Fe(III) complexation increased antimicrobial potency by approximately 10,000-fold. Similar trends were observed in MRSA^α^, where Fe(tropo)_3_ induced 1.5 log, 5.5 log, and 6.5 log reductions at 5.0, 10.0, and 20.0 µM, respectively (Figure 2b). These results demonstrate that coordination of Fe(III) to three lipophilic tropolone significantly enhances antibacterial efficacy against both MSSA and MRSA^α^ bacteria strains. However, MIC measurements against drug-resistant *Pseudomonas aeruginosa* (DRPA, ATCC BAA-2108) and *Acetobacter baumannii* (ATCC 19606) strains demonstrated that Fe(tropo)_3_ was inactive against this Gram-negative bacterium, with MIC values > 128 μg/mL (Figure S7), showing that the complex does not exhibit broad-spectrum antimicrobial activity.

### 2.3. Cellular Uptake Studies

With such remarkable antibacterial activity in different strains of SA bacteria, it would be beneficial to elucidate the mechanism of action of this Fe(tropo)_3_. To study the cell membrane penetration of Fe(tropo)_3_, the dose-dependent cellular uptake assay in MRSA^α^ was carried out on cell lysates using atomic absorption spectrometry (AAS). Two different sub-inhibitory concentrations of the compound were incubated with the bacterial cells for six hours. The FeCl_3_-treated cells were used as a reference group, and the untreated cells were the control group. The results showed that bacterial cells treated with 5.0 µM of Fe(tropo)_3_ had more than double the intracellular iron level compared to untreated cells, while cells treated with 10.0 µM had a fivefold increase in intracellular iron. In contrast, cells treated with FeCl_3_ at the same concentrations did not show a significant increase in intracellular iron, as depicted in Figure 2c. The six-hour incubation ensured that most cells remained viable and capable of taking up the Fe(tropo)_3_ complex. We attribute the remarkable uptake of iron to the electrically neutral and highly lipophilic character of Fe(tropo)_3_.

### 2.4. Anti-Biofilm Activity Against MRSA^α^ Derived Biofilms

Given the potent growth-inhibitory properties of Fe(tropo)_3_ against MRSA^α^, further exploration was conducted to determine its potential anti-biofilm activity. Bacteria growing inside biofilms can occasionally be 10–1000 times more resistant to conventional antibiotics than their planktonic counterparts as a result of an increased percentage of persister cells with specialized defensive mechanisms [29,30,31]. Therefore, we conducted anti-biofilm assays to test whether Fe(tropo)_3_ possesses any activity against biofilms derived from MRSA^α^ bacteria. The LIVE/DEAD assay results showed vigorous bactericidal activity against the MRSA^α^-derived bacteria in biofilms. Specifically, inhibition of MRSA^α^ bacteria in biofilms began at concentrations of Fe(tropo)_3_ below the MIC, i.e., 0.5 µg/mL (0.25× MIC) and 1.0 µg/mL (0.5× MIC). In comparison, a significant portion of the bacterial population from the biofilm is killed with the concentration of 2.0 µg/mL (1× MIC). Also, the side views of the biofilms from the treatment group demonstrated that their thickness gradually decreased in a dose-dependent manner in comparison to that of the control group, showing the presence of a bacteriostatic effect to prevent the growth of biofilms (Figure 2d). Additionally, quantitative CFU-based assays demonstrated that the antibiofilm activity was dose-dependent, resulting in partial inhibition of established biofilms, with 18% inhibition at 0.5 µg/mL (0.25× MIC), 36% inhibition at 1.0 µg/mL (0.5× MIC), and 58% inhibition at 2.0 µg/mL (1× MIC), respectively (Figure 2f).

### 2.5. The Time–Kill Assay

The time–kill kinetics of Fe(tropo)_3_ were evaluated against MRSA^α^ by monitoring bacterial growth through optical density (OD_600_) measurements at multiple time points. OD_600_ is a well-established proxy for bacterial cell density and correlates with viable cell counts (CFU/mL), particularly during logarithmic growth when light scattering by cells reflects changes in bacterial biomass [32]. Bacteriostatic activity was observed at concentrations ≤ 1× MIC, whereas bactericidal activity occurred at concentrations ≥ 2× MIC. At bactericidal concentrations, killing was relatively slow, with a significant reduction in OD_600_ detected only after 6 h or longer of treatment (Figure 2f). Therefore, the observed decline in OD_600_ at higher concentrations of Fe(tropo)_3_ supports a reduction in viable bacteria consistent with bactericidal activity.

### 2.6. Measurements of Intracellular Generation of Reactive Oxygen Species

**Figure 3:**
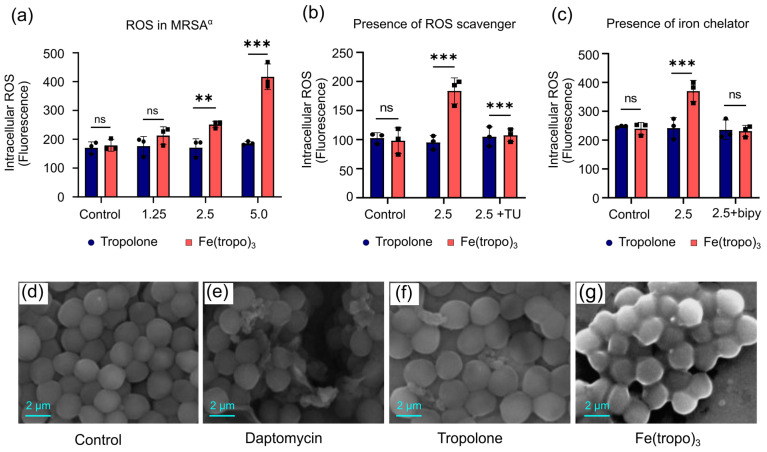
Tropolone and Fe(tropo)_3_ induced ROS production and morphological effects in MRSA^α^. (**a**) Normalized Intracellular ROS levels after 1 h treatment with increasing concentrations of tropolone or Fe(tropo)_3_. (**b**,**c**) ROS inhibition by thiourea (TU) and bipyridine (bipy), respectively. (**d**–**g**) SEM images of MRSA^α^: untreated control (**d**), daptomycin-treated (**e**), tropolone-treated (**f**), and Fe(tropo)_3_-treated (25 μM) (**g**). Data are shown as mean ± SD (*n* = 3 independent biological replicates). Statistical significance was determined as follows: ** *p* < 0.01, *** *p* < 0.001; ns, not significant.

The antibacterial activity of Fe is primarily attributed to its catalytic role in the Fenton reaction, which generates reactive oxygen species (ROS) within bacterial cells. We determined the intracellular levels of ROS in MRSA^α^ bacterial cells treated with different concentrations of Fe(tropo)_3_ and tropolone as the reference group using fluorescence-based DCFH-DA assay. The results demonstrate that treatment of MRSA^α^ with increasing concentrations of Fe(tropo)_3_ caused a dose-dependent increase in intracellular ROS, whereas tropolone alone had no effect, indicating that ROS generation by the Fe center plays a major role in bacterial killing (Figure 3a).

To validate the contribution of ROS to bacterial killing, we performed a rescue experiment with thiourea (TU), an ROS scavenger that mitigates intracellular ROS accumulation. TU effectively prevented ROS-mediated damage in MRSA^α^, fully restoring bacterial viability (Figure 3b). Additionally, the treatment of 2,2′-bipyridine (bipy), an Fe-chelating agent, was able to fully suppress intracellular ROS formation, confirming the dependence of bacterial killing consistent with Fe-mediated ROS generation (Figure 3c).

### 2.7. Scanning Electron Microscopic Imaging of Bacterial Cells

Scanning electron microscopy (SEM) was used to examine the morphology of bacterial cells treated with 25 µM of tropolone, Fe(tropo)_3_, or daptomycin, compared to untreated cells. The results show that daptomycin, a lipopeptide antibiotic that targets the bacterial cell membrane, caused membrane cleavage and cytosolic debris [33], while the cells treated with tropolone are wrapped with an unidentified thick shroud [34]. In contrast, Fe(tropo)_3_ treatment induced membrane damage leading to cell lysis, characterized by prominent membrane blebbing and leakage of intracellular contents. These observations indicate that Fe(tropo)_3_ disrupts bacterial membranes via a mechanism distinct from tropolone and daptomycin, consistent with the known deleterious effects of ROS on biological systems (Figure 3d–g) [27,35,36].

### 2.8. In Vitro Evaluation of Resistance Development

**Figure 4:**
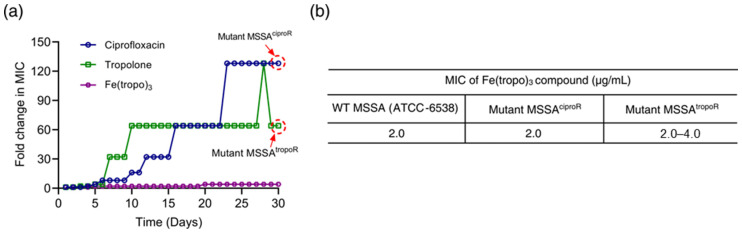
Results of in vitro assays of resistance development in MSSA toward Fe(tropo)_3_ in comparison with tropolone and ciprofloxacin (**a**); MIC values of Fe(tropo)_3_ against MSSA that become resistant to ciprofloxacin (mutant MSSA^ciproR^) and tropolone (mutant MSSA^tropoR^) (**b**).

Our results suggest that bacterial cell death induced by Fenton reaction-driven ROS signaling is the primary mechanism of action of Fe(tropo)_3_. We therefore hypothesized that Fe(tropo)_3_ might circumvent the resistance mechanisms typically observed with conventional antibiotics such as ciprofloxacin. We exposed MSSA to sub-lethal concentrations of Fe(tropo)_3_ over 30 successive passages and compared the results with those obtained for ciprofloxacin and tropolone. The initial MIC values of ciprofloxacin, tropolone, and Fe(tropo)_3_ against MSSA were 0.25, 80.0, and 2.0 μg/mL, respectively. Following serial passages, ciprofloxacin’s MIC increased progressively 8-fold after 6 passages, 16-fold at the 10th, 32-fold at the 12th, 64-fold at the 16th, and 128-fold by the 23rd passage. The MIC of tropolone increased 4-fold after 5 passages and then surged to 32-fold and 64-fold at the 7th and 10th passages, reflecting the emergence of a tropolone-resistant MSSA mutant (MSSA^tropoR^). In contrast, the MIC of Fe(tropo)_3_ remained largely stable through the 20th passage, with only a modest 8-fold increase thereafter, and remained unchanged up to the 30th passage (Figure 4a). We further evaluated whether Fe(tropo)_3_ retained activity against MSSA mutants resistant to tropolone and ciprofloxacin. The MICs against MSSA^tropoR^ and MSSA^ciproR^ after 30 passages were comparable to the initial MIC of wild-type MSSA, at 2–4 μg/mL and 2 μg/mL, respectively (Figure 4b). These findings indicate that Fe(tropo)_3_ does not exhibit cross-resistance with strains resistant to ciprofloxacin or tropolone, suggesting that its cellular targets are very distinct from those of these conventional antibiotics.

### 2.9. Checkerboard Assays

**Figure 5:**
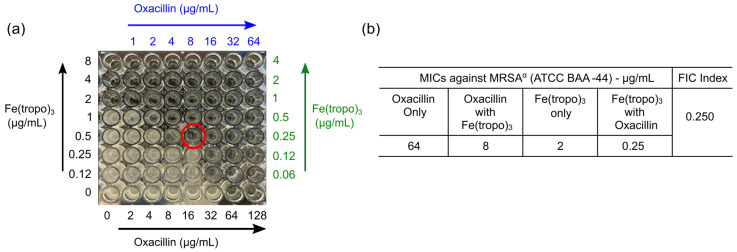
Checkerboard assay assessing the interaction between Fe(tropo)_3_ and oxacillin against MRSA^α^ (ATCC BAA-44). (**a**) Representative checkerboard microdilution plate showing bacterial growth inhibition across varying concentrations of Fe(tropo)_3_ and oxacillin, and (**b**) summary of minimum inhibitory concentrations (MICs) and fractional inhibitory concentration index (FICI) values derived from the checkerboard assay. The MICs of Fe(tropo)_3_ and oxacillin when tested alone were 2.0 µg/mL and 64 µg/mL, respectively. In combination, the MIC of oxacillin decreased to 8 µg/mL in the presence of 0.25 µg/mL Fe(tropo)_3_ (red circled-well), corresponding to FIC values of 0.125 for each agent and a combined FICI of 0.25, indicating synergistic activity.

Since the antibacterial mechanism of Fe(tropo)_3_ is distinct from those of conventional antibiotics, we next examined whether Fe(tropo)_3_ could potentiate the activity of oxacillin against MRSA^α^ using a checkerboard microdilution assay [37]. When tested individually, the MIC values of Fe(tropo)_3_ and oxacillin against MRSA^α^ were determined to be 2.0 µg/mL and 64 µg/mL, respectively (Figure 5). In the combination assay, the MIC of oxacillin was reduced eightfold to 8 µg/mL in the presence of 0.25 µg/mL Fe(tropo)_3_. The corresponding fractional inhibitory concentration (FIC) values were 0.125 for Fe(tropo)_3_ and 0.125 for oxacillin, yielding a combined FICI of 0.25. According to established interpretive criteria, this result indicates a synergistic interaction between Fe(tropo)_3_ and oxacillin against MRSA^α^. These findings suggest that Fe(tropo)_3_ may restore or enhance the efficacy of β-lactam antibiotics against drug-resistant *S. aureus* through a complementary, mechanism-distinct mode of action.

### 2.10. In Vitro Antimicrobial Effects of Mupirocin, Fusidate, and Fe(tropo)_3_ in Ointment Form

Since Fe(tropo)_3_ acts via a mechanism distinct from the molecular targets of conventional antibiotics, it holds promise as a potential alternative to mupirocin and fusidate for treating SSTIs caused by both MSSA and MRSA. To evaluate this possibility, we formulated PEG-based ointments containing either 1% Fe(tropo)_3_, 2% mupirocin, 2% fusidate, or DMSO as a vehicle control (Figure S8) and assessed their antibacterial activity using diffusion plate assays. These assays were performed against an MRSA^α^ strain susceptible to mupirocin and fusidate, as well as a high-level resistant MRSA^α^ strain, allowing direct comparison of the efficacy of Fe(tropo)_3_ relative to standard treatments (Figure S5).

**Figure 6:**
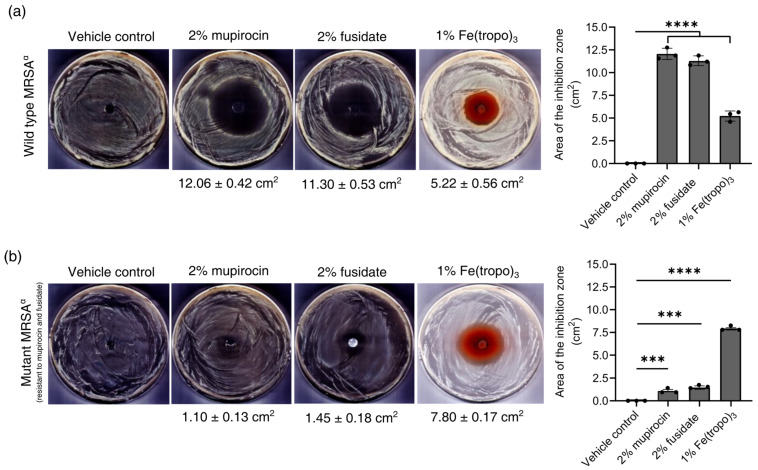
Antimicrobial zone of growth inhibition measures. The average zones of inhibition of PEG-based ointments containing the indicated antibiotics or 1% Fe(tropo)_3_ toward *S. aureus* strain (wild type MRSA^α^) (**a**) and high-level mupirocin and fusidate resistance MRSA^α^ [MRSA^α(mupR/fusR)^] (**b**). (mean ± s.d, *n* = 3 biological replicates; *** *p* < 0.001, **** *p* < 0.001).

As shown in Figure 6a, measures of each treatment’s zone of inhibition revealed that while vehicle control alone did not affect MRSA^α^ growth, both antibiotics and Fe(tropo)_3_ produced zones of growth inhibition, indicating that neither agent’s antibacterial properties were antagonized by the ointment formulation. More specifically, 2% mupirocin generated a zone of inhibition of 12.06 ± 0.42 cm^2^, whereas 2% fusidate exhibited an average zone of clearance of 11.30 ± 0.53 cm^2^. However, the 1% Fe(tropo)_3_ ointment treatment displayed a significantly lower inhibition zone (5.22 ± 0.56 cm^2^) compared to the zone of inhibition caused by mupirocin or fusidate alone.

As shown in Figure 6b, evaluation of the high-level mupirocin- and fusidate-resistant MRSA^α^ strain showed that both 2% mupirocin and 2% fusidate ointments produced small but detectable zones of inhibition (1.10 ± 0.13 cm^2^ and 1.45 ± 0.18 cm^2^, respectively), despite the strain’s resistance to these agents, indicating that the tested concentrations partially overcame the resistance phenotype. Notably, treatment with 1% Fe(tropo)_3_ ointment resulted in a substantially larger zone of inhibition (7.80 ± 0.17 cm^2^) compared with either agent alone. These findings indicate that while 1% Fe(tropo)_3_ is less potent than conventional antibiotics in strains susceptible to mupirocin and fusidate, its major advantage is evident in resistant strains, where it markedly outperforms the standard treatments. Overall, the ointment formulation preserves the activity of all tested agents, and Fe(tropo)_3_ represents a promising alternative for treating infections caused by MRSA strains resistant to both mupirocin and fusidate.

### 2.11. In Vivo Validation of Therapeutic Efficacy of 1% Fe(tropo)_3_ Ointment in a Murine Model with Excisional Wound Infection by S. aureus

**Figure 7:**
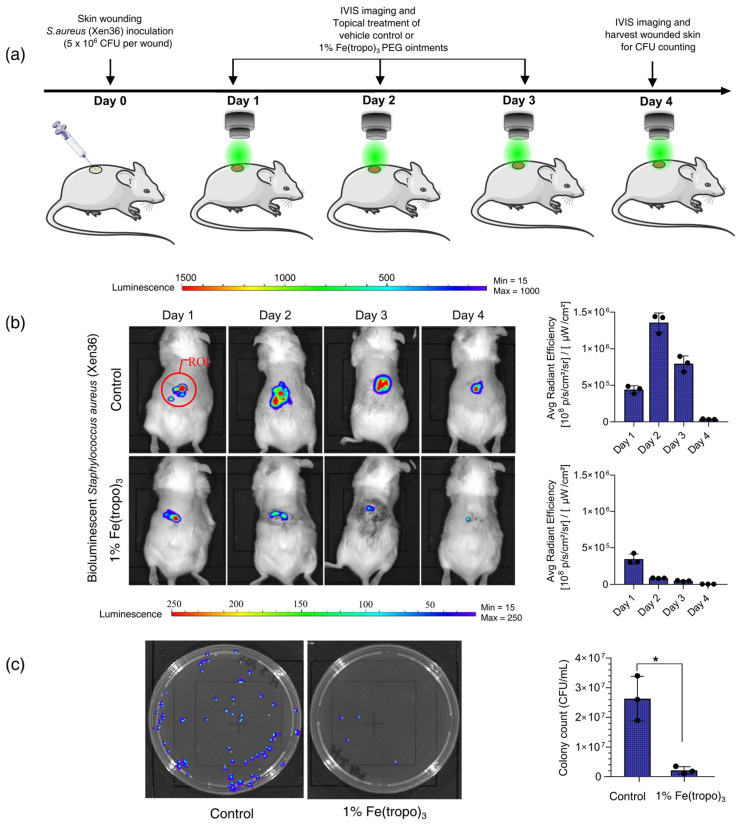
Therapeutic efficacy in a murine excisional wound infection model using *S. aureus* Xen36. (**a**) Schematic overview of the in vivo experimental design. (**b**,**c**) In vivo bioluminescence imaging of infected wounds with corresponding region-of-interest (ROI) quantification and bacterial burden (CFU) analysis following treatment. Data are shown as mean ± SD (*n* = 3 mice per group). Statistical significance was determined as follows: * *p* < 0.05.

We determined the in vivo therapeutic efficacy of 1% Fe(tropo)_3_ ointment using a mouse model of cutaneous wound infection with bioluminescent *S. aureus* (Xen36, PerkinElmer) (Figure S9). Jax Swiss outbred mice were divided into treatment and vehicle control groups and inoculated with 5 × 10^6^ CFU/mL of *S. aureus* in wounded skin on day 0. Bioluminescence imaging (IVIS) was performed 24 h post-inoculation to monitor infection. Mice then received daily topical applications of 50 µL of 1% Fe(tropo)_3_ or vehicle (DMSO/PEG) for three consecutive days. Wound lesion size and in vivo bacterial burden were evaluated through bioluminescence, and viable bacterial CFUs in wound tissues were quantified on day 4. Treatment with 1% Fe(tropo)_3_ significantly reduced lesion size and bioluminescent signals compared with the vehicle control and decreased bacterial burden by 98 ± 1% (Figure 7). Importantly, mice treated with Fe(tropo)_3_ showed no weight loss, indicating that the compound effectively lowers bacterial load without causing severe side effects, demonstrating its therapeutic potential for *S. aureus* skin infections.

### 2.12. Selectivity of Fe(tropo)_3_ Against MRSA

**Figure 8:**
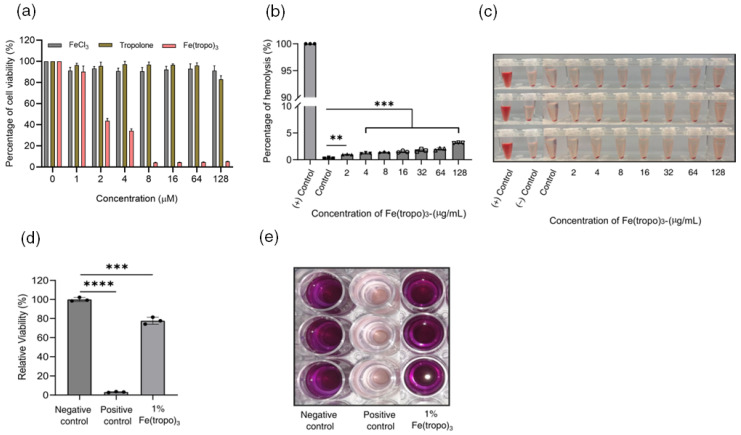
Evaluation of the cytotoxicity to mammalian cells, hemocompatibility, and skin irritation potential of Fe(tropo)_3_. (**a**) Cytotoxicity of FeCl_3_, tropolone, and Fe(tropo)_3_ on HDF cells. (**b**) Hemolysis percentages of Fe(tropo)_3_ at various concentrations. (**c**) Representative images of human red blood cells (hRBCs) after treatment with Fe(tropo)_3_. (**d**) Relative viability of reconstructed human epidermal tissue (EpiDerm) following exposure to 5% SDS (positive control) and 1% Fe(tropo)_3_ ointment compared with the negative control. (**e**) Representative images from the skin irritation assay. Data are shown as mean ± SD (*n* = 3 independent biological replicates). Statistical significance was determined as follows: ** *p* < 0.01, *** *p* < 0.001, **** *p* < 0.0001.

The cytotoxic effects of Fe(tropo)_3_ were investigated in human dermal fibroblast (HDF) cells using an MTT assay across concentrations ranging from 0 to 128 µM. Tropolone and FeCl_3_ were tested as reference compounds. Neither FeCl_3_ nor tropolone exhibited cytotoxicity within the examined concentration range (IC_50_ > 128 µM). In contrast, Fe(tropo)_3_ showed dose-dependent toxicity toward HDF cells, with IC_50_ values of approximately 2 µM (Figure 8a). A modest increase in cell viability was observed following FeCl_3_ treatment, suggesting a potential proliferative effect of iron on HDF cells. The selectivity of Fe(tropo)_3_ toward MRSA was assessed by calculating the selectivity index (S.I. = IC_50_/MIC). Fe(tropo)_3_ exhibited a selectivity index of 0.4, indicating toxicity toward mammalian cells rather than antimicrobial potency against MRSA. These preliminary in vitro toxicity results suggest that Fe(tropo)_3_ has a narrow therapeutic window, which may limit its suitability for systemic administration as an antimicrobial agent. The relatively low selectivity is likely attributable to oxidative stress arising from reactive oxygen species generated in mammalian cells upon exposure to the Fe(tropo)_3_ complex. To further assess the selectivity of Fe(tropo)_3_ as a potential antimicrobial agent, we evaluated its hemolytic activity in human red blood cells (hRBCs) using a previously described method [38]. Fe(tropo)_3_ exhibited minimal hemolytic activity toward human red blood cells, with an HC_50_ > 128 µg/mL and <5% hemolysis even at the highest concentration tested (Figure 8b,c). These results demonstrate that, despite its cytotoxic effects in mammalian cells, Fe(tropo)_3_ shows good hemocompatibility, highlighting a favorable safety profile toward erythrocytes.

### 2.13. In Vitro Skin Irritation Test

Assessing the potential of a drug substance to cause skin irritation is a critical aspect of developing a topical antimicrobial drug. The skin irritation potential of the Fe(III) complex was evaluated by topically exposing a reconstructed epidermis model to a 1% Fe(tropo)_3_ ointment, with a 5% sodium dodecyl sulfate (SDS) solution used as the positive control and water-treated tissues serving as the negative control. The model closely resembles the well-differentiated layers of the human epidermis, including the basal, spinous, and granular layers, as well as the cornified epidermal layer. By comparing the reduction in tissue viability of test compounds 1% Fe(tropo)_3_ and 5% SDS to that of negative controls, the skin irritation potential of Fe(tropo)_3_ ointment was calculated. As illustrated in Figure 8d,e, tissue viability was normalized to the negative control (100%). Treatment with the 1% Fe(tropo)_3_ ointment preserved most of the tissue viability (80 ± 3%), demonstrating greater skin compatibility, whereas exposure to 5% SDS resulted in extensive tissue damage, with viability dropping to 3 ± 1%, confirming its strong irritant effect on skin. These results indicate that the PEG-based ointment formulation of Fe(tropo)_3_ cytotoxicity by enabling controlled release and localizing Fe(tropo)_3_ at the wound site. The semi-solid, viscous PEG matrix confines the Fe(tropo)_3_ to the skin surface, limiting penetration into deeper tissues and systemic exposure, thereby minimizing skin irritation and making it a promising candidate for topical antimicrobial therapy.

## 3. Materials and Methods

All chemicals and solvents were obtained from commercial suppliers and used without further purification. FeCl_3_, tropolone, ethanol, nitric acid (HNO_3_), hydrochloric acid (HCl), dimethyl sulfoxide (DMSO), polyethylene glycol (PEG; MW 400 and PEG 3350), 2,2′-bipyridine (bipy), and thiourea (TU) were purchased from MilliporeSigma (Burlington, MA, USA). Tryptic Soy Broth (BD 211825), Tryptic Soy Agar (BD 236950), Luria–Bertani (LB) broth, and LB agar powder were obtained from Fisher Scientific. Ciprofloxacin (≥98%), oxacillin (≥98%), mupirocin (≥98%), and fusidic acid (≥98%) were also purchased from Fisher Scientific.

The Gram-positive bacterial strains used in this study—*Staphylococcus aureus* (ATCC 6538), methicillin-resistant *S. aureus* (ATCC BAA-44, USA300, ATCC BAA-1717), and vancomycin-intermediate *S. aureus* (VISA, ATCC 700699) and Gram-negative drug-resistant *P. aeruginosa* (DRPA, ATCC BAA-2108) and *Acetobacter baumannii* (ATCC 19606)—were obtained from the American Type Culture Collection. The bioluminescent *S. aureus* Xen36 strain was purchased from PerkinElmer (Shelton, CT, USA). The EpiDerm in vitro 3D human skin tissue model was obtained from MatTek Life Sciences, Ashland, MA, USA.

### 3.1. Synthesis and Characterization of Fe(tropo)_3_

First, tropolone (3.0 mmol) was dissolved in a 10 mL ethanolic solution in a 50 mL beaker. After iron (III) chloride (1.0 mmol) in 5 mL of ethanol was added to the reaction mixture, it was stirred for 3 h. The dark red-purple-colored suspension was then filtered to collect the crude product, washed with ethanol, and dried in a vacuum oven. The purity of the Fe(tropo)_3_ product was determined by elemental analysis. Further, the product was characterized by UV-Vis, FT-IR, and X-ray powder diffraction (see the Supplementary Materials for details).

### 3.2. Minimum Inhibitory Concentration (MIC) Assays

The MICs of tropolone and Fe(tropo)_3_ against MSSA, MRSA, VISA, DSPA, and DRPA strains were determined according to CLSI guidelines, as described previously [28]. Briefly, bacterial suspensions (1 × 10^6^ CFU/mL) were treated with serial dilutions of each compound in 96-well plates, incubated for 24 h, and MICs were defined as the lowest concentrations showing no visible growth.

### 3.3. Investigation of Antibacterial Activity of Tropolone and Fe(tropo)_3_

*S. aureus* was grown in TSB at 37 °C with shaking (180 rpm) for 24 h, diluted 1:100 into fresh medium, and incubated for 4 h to reach ~1 × 10^9^ CFU/mL. The culture was then diluted to 1 × 10^6^ CFU/mL, treated with tropolone or Fe(tropo)_3_ in DMSO, and incubated for 24 h [39]. Viable bacteria were quantified by CFU counting on agar plates.

### 3.4. Determination of Intracellular Iron Content

The cellular uptake of Fe(tropo)_3_ was examined by quantifying the amount of Fe inside the bacteria using AAS, as reported previously [27,40,41,42]. First, two different doses of 5.0 μM and 10.0 μM of Fe(tropo)_3_ or FeCl_3_ were used to treat bacterial cells and incubated for 6 h. The bacterial cells were collected by centrifuging and washed with deionized water. After that, the bacterial cell pellets were digested with 75% HNO_3_ and the sample was calcined at 620 °C for 5 h. The residual content was dissolved in 1 mL of aqua regia, and the Fe contents were determined by AAS.

### 3.5. Time–Kill Assay

We performed the time–kill assays against MRSA^α^ using a procedure as described previously [42]. Briefly, early exponential phase-grown bacteria were treated with several concentrations of Fe(tropo)_3_, and the optical density at 600 nm was measured at selected time intervals.

### 3.6. Biofilm Inhibition Assay

The inhibition of biofilm assay was performed using a previously described method [43]. Briefly, MRSA^α^ bacteria treated with Fe(tropo)_3_ were grown in an 8-well plate. Then, the plate was incubated for 24 h at 37 °C to form biofilms and washed with phosphate-buffered solution (PBS, 1×). After, 200 μL of 0.3% SYTO9 and PI (propidium iodide) stains in PBS (LIVE/DEAD BacLight Bacterial Viability Kit, Thermo Fisher Scientific, Waltham, MA, USA) was added to each biofilm. Then the plate was incubated for 20 min. Finally, sample wells were washed with PBS 3 times, and an inverted fluorescence microscope (Olympus IX81, Tokyo, Japan) was used to capture the images.

### 3.7. Bacterial Reactive Oxygen Species (ROS) Measurements

Intracellular ROS levels in MRSA^α^ were measured as described previously [27]. Briefly, bacteria were treated with varying concentrations of tropolone or Fe(tropo)_3_ and incubated at 37 °C for 1 h with shaking. Cells were then harvested, washed with 1× HBSS, and incubated with 20 μM DCFH-DA for 30 min at 37 °C in the dark. Fluorescence was measured using a SpectraMax, San Jose, CA, USA, M4 microplate reader at 497/529 nm (excitation/emission).

### 3.8. Measurements of Intracellular ROS Scavenging Effect

Bacterial cells were treated with Fe(tropo)_3_ in the presence of thiourea TU (200 mM) or 2,2′-bipyridine (250 mM) and in the absence of thiourea or 2,2′-bipyridine as a comparison. After, the bacterial cells were collected by centrifugation and rinsed twice with Hank’s balanced salt solution (HBBS 1×). The bacterial suspensions were incubated with 20 μM DCFH-DA at 37 °C with agitation for 30 min. The fluorescence intensity of bacteria was measured by a microplate reader (SpectraMax M4) at 497/529 nm (excitation/emission).

### 3.9. Visualization of Cell Membrane Integrity by SEM

The morphology of MRSA^α^ bacterial samples was monitored using a Quanta450 Field Emission Gun Scanning Electron Microscope (FEI, Waltham, MA, USA) using a previously reported procedure [27,41,44,45]. Briefly, MRSA^α^ (1 × 10^9^ CFU/mL) was treated with tropolone, daptomycin, and Fe(tropo)_3_ (all at 25 μM) for 2 h. The bacterial suspension was centrifuged at 3750 rpm and washed with phosphate-buffered solution (PBS 1×). Bacterial cells were then fixed in 2.5% glutaraldehyde (in PBS) and treated with 1% tannic acid for 10 min. After fixation, the cells were washed with 1 mL of PBS and dehydrated using a series of ethanolic solutions (25%, 30%, 50%, 75%, and 100%, respectively). Finally, dried bacterial samples were coated with gold before the SEM (HR-SEM) imaging studies.

### 3.10. Checkerboard Assay

The checkerboard assay was performed as described previously [27,46]. Oxacillin and Fe(tropo)_3_ were twofold serially diluted along the row and column axes, respectively, to generate a concentration matrix. Wells were inoculated with MRSA^α^ (ATCC BAA-44) to a final density of ~1 × 10^6^ CFU/mL in 100 μL and incubated at 37 °C for 20 h. MICs were determined by visual inspection. Fractional inhibitory concentrations (FICs) were calculated as the MIC of each agent in combination divided by its MIC alone, and the FIC index (FICI) was obtained by summing both FICs. Interactions were defined as synergistic (χ ≤ 0.5), additive (0.5 < χ < 4), or antagonistic (χ ≥ 4) [37].

### 3.11. In Vitro Assay for Resistance Development

Resistance development assays were performed by successive passaging of bacteria as previously described [27,44,47]. MSSA was serially exposed to tropolone, Fe(tropo)_3_, or ciprofloxacin for 30 days. After each 24 h incubation at 37 °C, bacteria grown at the highest drug concentration were collected and the MIC was re-determined. This process was repeated for 30 passages, and resistance development was expressed as the fold change in MIC relative to day 1.

### 3.12. Preparation of High-Level Mupirocin and Fusidate-Resistant MRSA Strains of SA Bacteria

The resistance development by serial passaging was carried out as reported previously [27,41,44]. First, the potential for developing mupirocin resistance was evaluated by serial exposure of antibiotics to wild-type MRSA^α^ (WT MRSA^α^) for 50 consecutive days. After 24 h incubation at 37 °C, bacterial cells grown at the highest concentration of mupirocin were once again harvested and assayed for determination of MICs. This process was continuously repeated for 50 passages until the wild-type MRSA^α^ became resistant to mupirocin (MIC ≥ 2.0 mg/mL). Second, mutant bacteria, resistant to mupirocin, were treated with fusidate continuously for another 70 passages until they became resistant to both antibiotics: mupirocin and fusidate (MIC ≥ 2.0 mg/mL). Results were expressed as a fold increase in MIC with respect to the MIC on day 1 (Figure S5).

### 3.13. Preparation of 2% Mupirocin, 2% Fusidate and 1% Fe(tropo)_3_ PEG Based Ointment

To prepare the polyethylene glycol (PEG) ointment base PEG 400 (70%, wt/vol) was mixed with PEG 3350 (30%, wt/vol) according to the guidelines of U.S. Pharmacopeia and The National Formulary (USP 24-NF 19) [48]. Thereafter, 2.5 g of PEG ointment base was combined with 50 mg of mupirocin and fusidate that had been dissolved in 250 µL of DMSO while the solution was kept at 60 °C to create a homogeneous solution that contained 2% mupirocin and 2% fusidate. Those solution mixtures were then allowed to cool to room temperature with stirring to solidify. Similarly, 1% Fe(tropo)_3_ ointment was prepared by adding 25 mg of Fe(tropo)_3_ (in 250 µL of DMSO) to the 2.5 g of PEG ointment base (Figure S7).

### 3.14. In Vitro Ointment Antimicrobial Testing

Inhibition of antimicrobial zones was evaluated for PEG ointments using the MRSA strains reported previously with modifications [48]. Briefly, 100 µL of 1 × 10^8^ CFU/mL of MRSA was spread on tryptic soy agar (TSA) plates. Then, 40 µL of pre-liquified ointment was transferred onto the center of the agar plate. The plates were incubated at 37 °C for 24 h, and ImageJ software (version 1.53) was used to calculate the area of the inhibitory zone.

### 3.15. Excisional Murine Skin Wound Infection Model with S. aureus

All animal procedures were carried out in accordance with the Guide for the Care and Use of Laboratory Animals and were given the Institutional Animal Care and Use Committee’s (IACUC) approval at Northeast Ohio Medical University’s Department of Pharmaceutical Sciences in the College of Pharmacy. Male and female Jax Swiss outbred mice (8–12 weeks old) were obtained from Jackson Laboratory and used to establish a local wound infection model, as previously described. Mice were anesthetized with isoflurane, the dorsal skin was shaved and disinfected, and a 5 mm full-thickness wound was created and covered with Tegaderm. The wound was inoculated with 40 µL of bioluminescent *S. aureus* (Xen36; 5 × 10^6^ CFU/mL). At day 1 post-infection, IVIS was performed, followed by daily topical treatment with 1% Fe(tropo)_3_ (50 µL) or vehicle for 3 days. On day 4, wounds were excised and homogenized, and bacterial burden was quantified by CFU plating and IVIS, excluding non-luminescent colonies (Figure S9).

### 3.16. Cytotoxicity to the Mammalian Cells

The cytotoxicity of Fe(tropo)_3_, tropolone, and FeCl_3_ was evaluated in the human dermal fibroblast cell line using the MTT assay. Briefly, cells were seeded in 96-well plates (2 × 10^5^ cells/mL, 100 μL/well) and incubated for 24 h at 37 °C in 5% CO_2_. The medium was replaced with fresh medium containing various concentrations of the compounds, followed by a 24 h incubation. MTT reagent (10 μL) was added, and plates were incubated for 2 h at 37 °C. Formazan crystals were dissolved with 100 μL DMSO, and absorbance was measured at 570 nm. Experiments were performed in triplicate, and IC_50_ values were calculated using GraphPad Prism. Cell viability was expressed relative to untreated controls.

### 3.17. Hemolysis Assay

The hemolytic activity was carried out as reported previously [38,49]. Briefly, human blood cells (hRBCs) were subjected to various concentrations of Fe(tropo)_3,_ resulting in final concentrations of 4, 8, 16, 32, 64, or 128 μg/mL as the test group, while distilled water and DPBS were employed as the positive control and negative control group, respectively. Samples were incubated for 4 h at room temperature, followed by centrifugation for 5 min at 10,016 g. Thereafter, 100 μL of the supernatant from each sample was added to a 96-well plate, and a microplate reader was used to measure the absorbance at 577 nm with a reference wavelength of 655 nm. The hemolysis ratio was calculated using the formula hemolysis ratio = (OD_(test)_ − OD_(negative control)_)/(OD_(positive control)_ − OD_(negative control)_) × 100% to represent the degree of hemolysis.

### 3.18. In Vitro Skin Irritation Assay

An in vitro skin irritation assay was performed using the EpiDerm™ epidermal model (MatTek) according to the manufacturer’s protocol. Tissue inserts were pre-incubated at 37 °C and 5% CO_2_, transferred to fresh medium, and incubated overnight. The tissues were then topically treated with 30 µL of 1% Fe(tropo)_3_ ointment, 5% SDS (positive control), or PBS (negative control) for 1 h, followed by thorough rinsing with PBS. The inserts were incubated in fresh medium for 24 h and then post-incubated for an additional 18 h. Tissue viability was assessed using the MTT assay by incubating the tissues with MTT for 3 h, extracting the formazan with isopropanol overnight, and measuring the absorbance at 570 nm using a SpectraMax^®^ M4 plate reader (Molecular Devices, San Jose, CA, USA). Cell viability was expressed as a percentage relative to the negative control.

### 3.19. Statistical Analysis

The statistical analysis was done with GraphPad Prism (version 8.0). At least three biological replicates were used in the in vitro tests. The statistical significance between the two groups was determined using a two-tailed unpaired *t*-test. After one-way ANOVA, the Holm–Sidak comparisons test was used to evaluate the statistical significance between groups. All data were expressed as mean and standard error, and statistical significance was assessed by a *p*-value of less than 0.05.

## 4. Conclusions

The results presented in this work indicate that the use of Fe(tropo)_3_ as a topical antibacterial agent against different strains of SA bacteria, be they susceptible or resistant to the conventional antibiotics including mupirocin and fusidate, is potentially a prudent replacement to reduce the probability of resistance development to mupirocin and fusidic acid—two topical antibiotics that are widely used to treat SSTIs by SA as well as frequently applied in clinics for SA decolonization. Notably, Fe(tropo)_3_ demonstrated potent activity against SA strains resistant to both mupirocin and fusidate. The antibacterial mechanism of Fe(tropo)_3_ involves reactive oxygen species (ROS) generation via a Fenton-type reaction. Initially, the Fe^3+^ center in Fe(tropo)_3_ is reduced to Fe^2+^ by intracellular reducing agents or microbial ferric reductases (e.g., carotenoids) [50]. Because Fe^2+^ is a softer Lewis acid than Fe^3+^, it shows a weaker affinity for the hard oxygen-donor atoms of tropolone, leading to dissociation of the metal ion from the ligand. The resulting coordinatively unsaturated Fe^2+^ reacts with intracellular H_2_O_2_ to produce highly reactive hydroxyl radicals via the Fenton reaction, leading to bacterial cell death. While the in vivo data are derived from a pilot mouse study and should be considered proof-of-concept, they warrant validation in larger, statistically powered studies to confirm therapeutic efficacy and clinical relevance. Although systemic use of Fe(tropo)_3_ may be limited by fibroblast cell (HDFs) cytotoxicity, the localized nature of topical delivery and minimal hemolytic activity suggest that Fe(tropo)_3_ is a strong candidate for further development as a topical antimicrobial agent.

## Figures and Tables

**Table 1:** MIC values of different ligands and their iron complexes against Gram-positive *S. aureus*.

Compound Name	MIC (µg/mL)	Ligand LogP
1,10-Phenanthroline	64	2.20
Fe(1,10-Phenanthroline)_3_	64	-
2,2′-bipyridine	>256	1.88
Fe(2,2′-bipyridine)_3_	>256	-
Hinokitiol	16	0.59
Fe(hinokitiol)_3_	2	-
Acetylacetone	256	0.24
Fe(Acetylacetone)_3_	256	-
Tropolone	80	−0.34
Fe(Tropolone)_3_	2	-
Deferiprone	>256	−0.83
Fe(Deferiprone)_3_	>256	-
Ethyl maltolate	>128	−1.11
Fe(Ethyl maltolate)_3_	64	-
Methyl maltolate	>256	−1.60
Fe(Methyl maltolate)_3_	128	-

**Table 2:** MIC values of tropolone and Fe(tropo)_3_ against Gram-positive SA strains of bacteria.

	Minimum Inhibitory Concentration (MIC) µg/mL	
Bacteria Strains	Tropolone	Fe(Tropo)_3_
MSSA (ATCC 6538)	80	2
MRSA^α^ (ATCC BAA-44)	160	2
MRSA^β^ (ATCC BAA-1717)	160	2
VISA (ATCC-700699)	160	2
MRSA^α(mupR)^	160	2
MRSA^α(fudR)^	160	2
MRSA^α(mupR+fusR)^	160	2

## Data Availability

The published article and its supplemental information files both contain all of the data generated or analyzed throughout this study, and they can both be accessed without charge.

## Supplementary Materials

The following supporting information can be downloaded at: https://www.mdpi.com/article/10.3390/antibiotics15030298/s1.

## Abbreviations

AMR	Antimicrobial Resistance
SSTIs	Skin and Soft Infections
CO-ADD	Community for Open Antimicrobial Drug Discovery
ROS	Reactive Oxygen Species
MSSA	Methicillin-Sensitive Staphylococcus aureus
MRSA	Methicillin-Resistant Staphylococcus aureus
MDR	Multidrug Resistant
DNA	Deoxyribonucleic Acid
hRBCs	Human Red Blood Cells
CFU	Colony Forming Unit
MIC	Minimum Inhibitory Concentration
FICI	Fractional Inhibitory Concentration Index
