# Supplementary material for: Iron(III)–Tropolone Complex as a Topical Agent Against Drug-Resistant MRSA Skin Infections
Source: Antibiotics (Basel). 2026 Mar 14;15(3):298. doi: 10.3390/antibiotics15030298 (PMC13023664; doi:10.3390/antibiotics15030298)
Supplement: Supplementary file 1 [file antibiotics-15-00298-s001.zip › antibiotics-4132545-supplementary.pdf]

Supporting Information

Iron(III)–Tropolone Complex as a Topical Agent against Drug-Resistant MRSA Skin Infections

Nalin Abeydeera[1], Guanyu Chen[1], Khalil Zarea[1,4], Bishnu D. Pant[1], Bogdan M. Benin[6], Kalpani M. Ratnayake[3], Min-Ho Kim[2], Woo Shik Shin*[5] and Songping D. Huang*[1]

List of Figures and Tables

Figure S1. Images of MIC evaluations for ligands and their corresponding Fe(III) complexes against MSSA (a-g).

Table S1. Results of elemental analysis for $Fe(tropo)_3$.

Scheme S1. Synthesis of $Fe(tropo)_3$ in ethanol at room temperature.

Figure S2. UV-Vis spectrum of $Fe(tropo)_3$ and tropolone in chloroform.

Figure S3. FT-IR spectra of $Fe(tropo)_3$ and tropolone.

Figure S4. Experimental and simulated X-ray powder diffraction patterns of $Fe(tropo)_3$.

Figure S5. Resistance development profile of mupirocin and fusidic acid in MRSA$^{\alpha}$ (ATCC BAA-44).

Figure S6. Images of MIC test for tropolone and $Fe(tropo)_3$ against different strains of SA (a-g).

Figure S7. Images of MIC test for $Fe(tropo)_3$ against Gram negative *Pseudomonas aeruginosa* (left) and *Acetobacter baumannii* (right).

Figure S8. Images of prepared PEG-based vehicle control, 2% mupirocin, 2% fusidic acid, and 1% $Fe(tropo)_3$.

Figure S9. IVIS imaging of SA (Xen36) used for CFU optimization prior to murine wound infection.

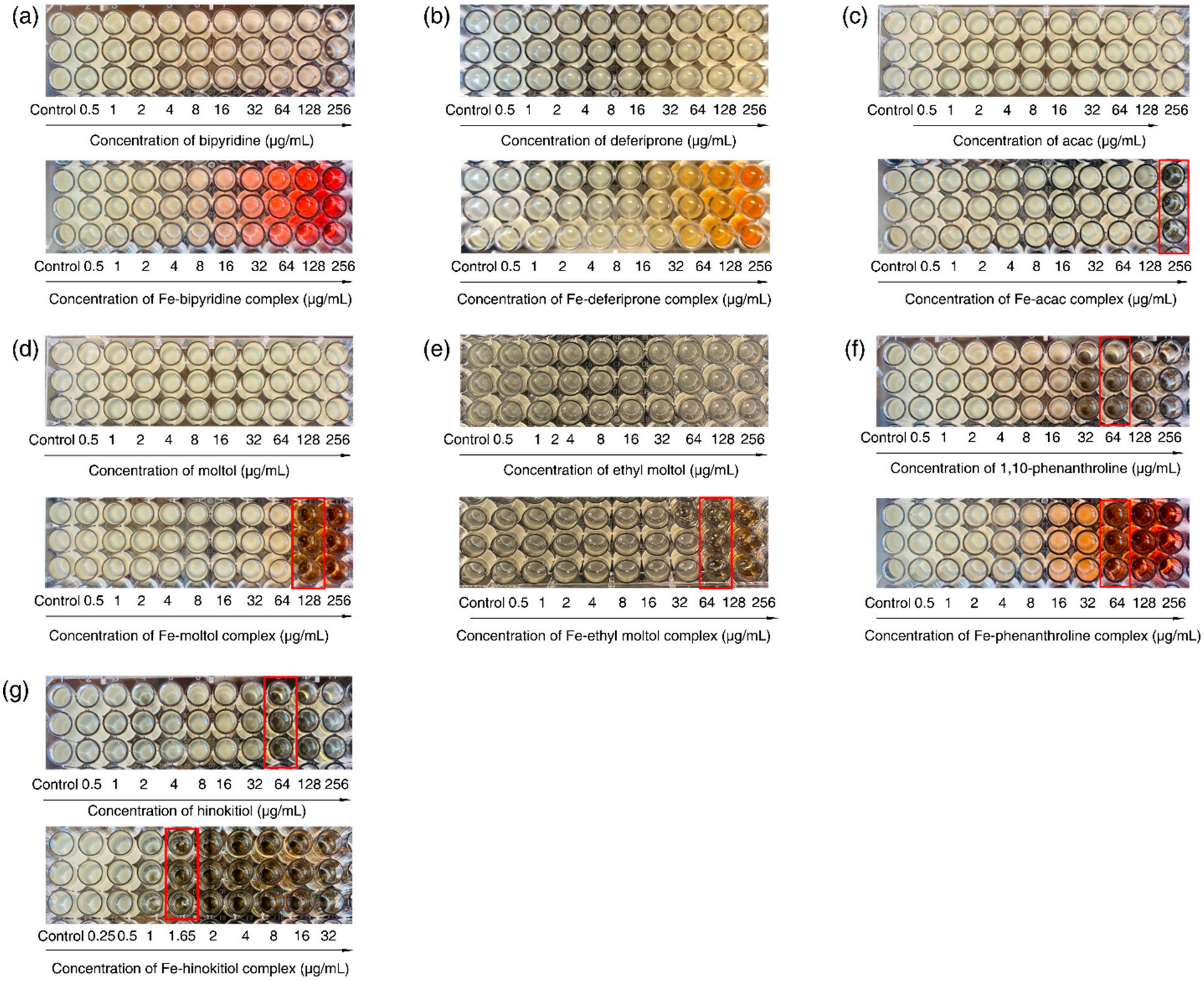

Figure S1. Images of MIC evaluations for ligands and various Fe(III) complexes against MSSA (a-g).

Table S1. Results of elemental analysis for Fe(tropo)$_3$*

$C_{21}H_{15}O_6Fe$	Calculated (%)	Found (%)
Percent C content	60.17	59.93
Percent H content	3.61	3.68
Percent Fe content	13.32	13.18

*Empirical formulas related to experimental values aided in obtaining the exact molecular formula of Fe(tropo)$_3$ complex. It can be seen that experimental values are within ± 0.3% of expected values indicating the purity of the synthesized complex is > 98%.

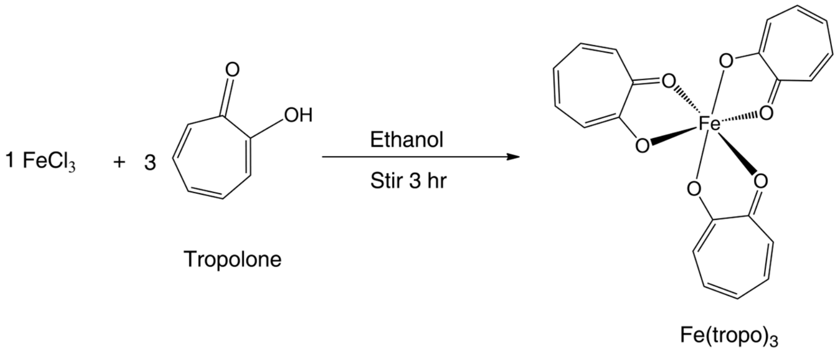

Scheme S1. Synthesis of Fe(tropo)3 in ethanol at room temperature.

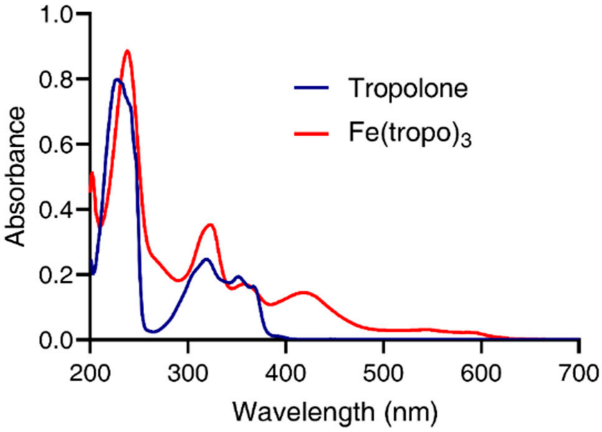

Figure S2. UV-Vis spectrum of Fe(tropo)3 and tropolone in chloroform. UV-Vis spectra of tropolone and Fe(tropo)3 complex were recorded in chloroform at room temperature. Tropolone has an aromatic ring system, and π–$\pi*$ transitions are thus possible. The absorption wavelengths of Fe(tropo)3 complex have shifted towards longer wavelengths (bathochromic shift) compared to the wavelengths of the tropolone.

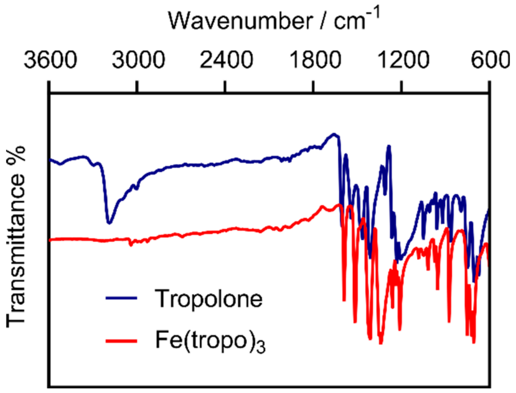

Figure S3. FT-IR spectra of $Fe(tropo)_3$ and tropolone. FT-IR data were recorded for dried powder of tropolone and $Fe(tropo)_3$ complex. The stretching frequency of bonds (C=O) and (C-O) are considered mostly because their values change upon the formation of new bonds serving as good indicators of complex formation. Stretching frequencies of (C=O) and (C-O) in $Fe(tropo)_3$ complex have shifted to lower frequencies as expected, compared to those values of the free tropolone ligand, due to σ donation of O lone pair which lowers the strength of (C=O) and (C-O) bonds. Furthermore, a broad band around 3400–3300 cm−1 of tropolone disappeared due to the deprotonation of the phenolic OH while forming the metal-ligand bond.

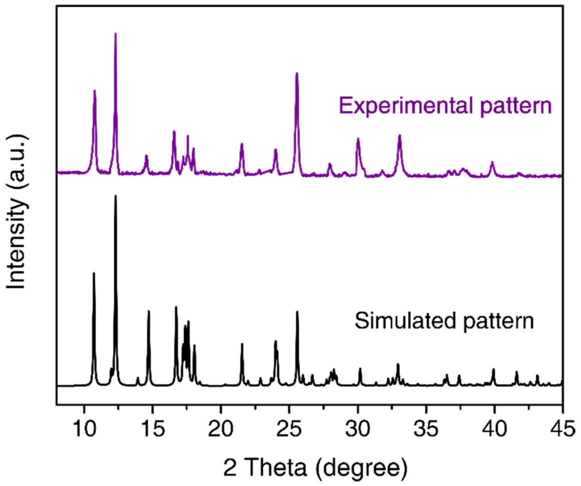

Figure S4. Experimental and simulated X-ray powder diffraction patterns of $Fe(tropo)_3$. Experimental X-ray powder diffraction pattern of the synthesized $Fe(tropo)_3$ complex gives the exact similar pattern that coincides with the simulated pattern and clearly confirms that the identity of this product was $Fe(tropo)_3$ with purity ≥ 98%.

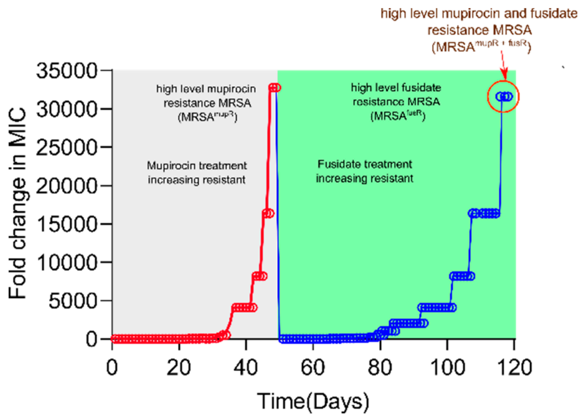

Figure S5. Resistance development profile of mupirocin and fusidic acid in MRSA$^{\alpha}$ (ATCC BAA-44).

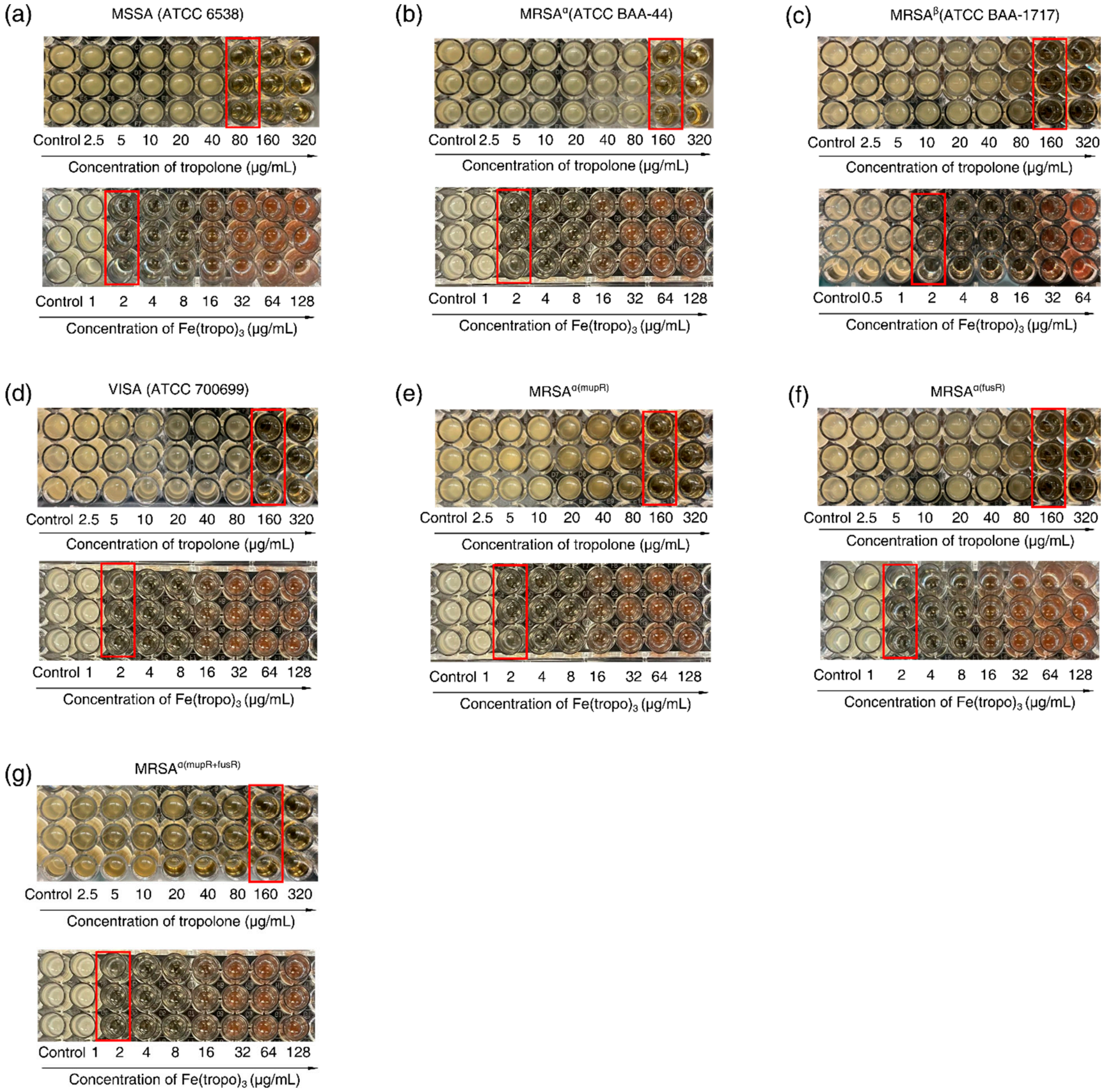

Figure S6. Images of MIC test for tropolone and $Fe(tropo)_3$ against different strains of SA (a-g).

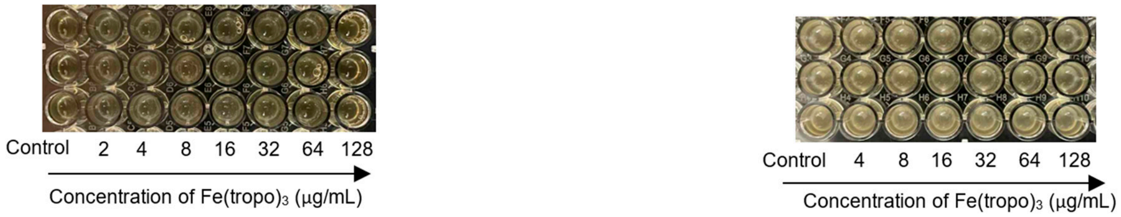

Figure S7. Images of MIC test for $Fe(tropo)_3$ against Gram negative *Pseudomonas aeruginosa* (left) and *Acetobacter baumannii* (right).

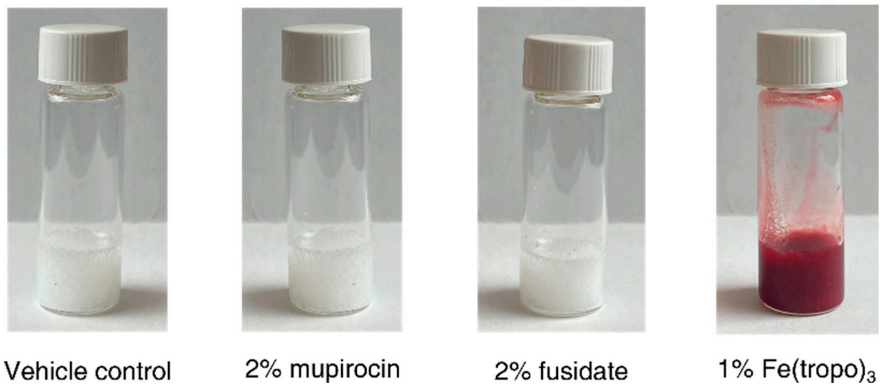

Figure S8. Images of prepared PEG-based vehicle control, 2% mupirocin, 2% fusidate, and 1% $Fe(tropo)_3$.

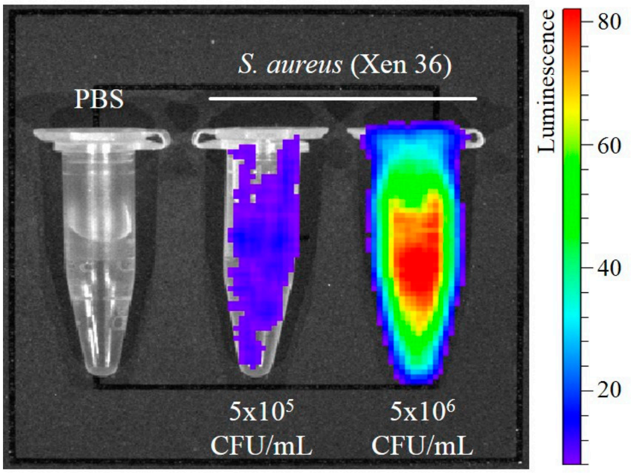

Figure S9. IVIS imaging of SA (Xen36) used for CFU optimization prior to murine wound infection.
